# The Role of SIRT3 in Exercise and Aging

**DOI:** 10.3390/cells11162596

**Published:** 2022-08-20

**Authors:** Lei Zhou, Ricardo Pinho, Yaodong Gu, Zsolt Radak

**Affiliations:** 1 Research Institute of Molecular Exercise Science, Hungarian University of Sport Science, H-1123 Budapest, Hungary; 2 Laboratory of Exercise Biochemistry in Health, Graduate Program in Health Sciences, School of Medicine, Pontifícia Universidade Católica do Paraná, Curitiba 80215-901, Brazil; 3 Faculty of Sports Science, Ningbo University, Ningbo 315211, China; 4 Faculty of Sport Sciences, Waseda University, Tokorozawa 359-1192, Japan

**Keywords:** SIRT3, exercise, metabolism, redox balance, aging

## Abstract

The health benefits of regular exercise are well established. Nonetheless, the molecular mechanism(s) responsible for exercise-induced health benefits remain a topic of debate. One of the key cell-signaling candidates proposed to provide exercise-induced benefits is sirtuin 3 (SIRT3). SIRT3, an NAD+ dependent mitochondrial deacetylase, positively modulates many cellular processes, including energy metabolism, mitochondrial biogenesis, and protection against oxidative stress. Although the exercise-induced change in SIRT3 signaling is a potential mechanism contributing to the health advantages of exercise on aging, studies investigating the impact of exercise on SIRT3 abundance in cells provide conflicting results. To resolve this conundrum, this narrative review provides a detailed analysis of the role that exercise-induced changes in SIRT3 play in providing the health and aging benefits associated with regular physical activity. We begin with an overview of SIRT3 function in cells followed by a comprehensive review of the impact of exercise on SIRT3 expression in humans and other mammalians. We then discuss the impact of SIRT3 on aging, followed by a thorough analysis of the cell-signaling links between SIRT3 and exercise-induced adaptation. Notably, to stimulate future research, we conclude with a discussion of key unanswered questions related to exercise, aging, and SIRT3 expression.

## 1. Introduction

Sirtuin3 (SIRT3) is an NAD-+-dependent protein deacetylase located mainly within the mitochondria. SIRT3 exerts control over a wide range of mitochondrial functions, including energy metabolism and protection against oxidative stress [1]. Moreover, SIRT3 expression is associated with protection against age-related cellular dysfunction [2]. Importantly, SIRT3 is the only sirtuin reported to affect human lifespan [3,4]. Therefore, increasing the expression and activity of SIRT3 has important implications for health and longevity.

SIRT3 is widely expressed in mitochondria-rich tissues with high metabolic demand, including the brain, heart, kidney, muscle, and brown adipose tissue [5]. Both caloric restriction and exercise have been reported to increase the expression of SIRT3 in various tissues [6]. While studies consistently conclude that caloric restriction increases the expression of SIRT3, studies on the impact of exercise on SIRT3 abundance in tissues are not universally consistent. Indeed, the impact of exercise on the abundance of SIRT3 in human and rodent tissues remains controversial with reports indicating that exercise does not systematically elevate SIRT3, whereas other studies conclude that regular bouts of endurance exercise do increase SIRT3 abundance in several tissues [7,8,9,10]. To resolve the confusion related to this important topic, this review provides a comprehensive summary of the available literature on the impact of exercise on SIRT3 in both human and animal tissues. The literature was surveyed using the PubMed database. The research used keywords: 1. SIRT3; 2. Exercise/Training/Treadmill/Running/Biking/HIIT/Swimming/Resistance Training/Voluntary Running/Endurance Training; 3. People/Subject/Human/Adult or Mice/Rats/Animal/Rodent/In Vitro. Then, we screened all available results and checked the citation of some key research. We included studies relating to the effect of exercise on SIRT3 expression in human and animals. Of notice, we did not use biased selection to exclude unfavorable data. Moreover, we examine the potential health benefits of increasing SIRT3 abundance and also discuss the biochemical connections between SIRT3 and exercise. We begin with a brief overview of the SIRT3 regulators, nicotinamide adenine dinucleotide (NAD) and NADH, and its subsequent signaling, the SIRTs family.

## 2. SIRT3: Big Picture Overview

The pyridine nucleotides, NAD, and NADH are coenzymes that provide oxidoreductive power for the generation of adenosine triphosphate (ATP) by mitochondria. Muscular exercise and other types of metabolic stimuli can impact cellular NAD and NADH levels. For example, exercise perturbs the levels of NAD+ and NADH in muscle fibers, and consequently, the NAD+/NADH ratio to fulfill the energy demand of contraction [11]. This is important because NAD+ levels and its downstream signaling are related to aging and age-related diseases. Specifically, NAD+ levels steadily decline with age, resulting in altered metabolism and increased disease susceptibility [12]. Conversely, restoration of NAD+ levels in old or diseased animals can promote health and extend lifespan [12]. Due to the widespread interest in understanding the biology of aging, numerous signaling pathways sensitive to NAD+(H) perturbations have received increased scientific interest. Much of the renewed interest in NAD over the last decade can be attributed to the Sirtuins (SIRTs), a family of NAD+-dependent protein deacetylases with remarkable abilities in promoting longevity and alleviating aging-associated diseases [13]. Meanwhile, similar to the exercise-sensing effect of NAD, SIRTs are also closely related to exercise [14]. In this context, the SIRTs family has received significant research attention.

SIRT was first discovered in yeast as “silent mating type information regulator 2” (first labeled as SIR2) in 1979 [15]; later, Frye discovered that mammalian SIRTs metabolize NAD+ in 1999 [16]. Later, Guarente and Sternglanz revealed that yeast SIR2 is an NAD+-dependent histone deacetylase [17]. Since then, SIRTs have been shown to play a major regulatory role in almost all cellular functions, including mitochondrial biogenesis, oxidative stress, inflammation, cell growth, circadian rhythms, energy metabolism, neuronal function, and stress resistance [12]. Currently, seven SIRTs have been discovered with different functions and cellular locations. SIRT1 and SIRT2 mainly exist in the nucleus and cytoplasm, while SIRT6 and SIRT7 are expressed in the nucleus. In addition, SIRT3, SIRT4, and SIRT5 are primarily expressed in the mitochondria, and SIRT3 is known as the main mitochondria deacetylase protein [18]. Notably, in the nucleus, SIRT3 also functions as the stabilizer of heterochromatin and antagonizer of cellular senescence in human mesenchymal stem cells [19]. Additionally, evidence indicates that SIRT3 can be transported from the nucleus to the mitochondria upon cellular stress [20], indicating the multifunction of SIRT3.

Again, SIRT3 is the only SIRT that positively impacts human aging, as numerous studies have linked SIRT3 to longevity in humans and rodents [13,21]. Over the past decade, there has been an expansion of research exploring the therapeutic potential of SIRT3 [1]. Exercise, commonly recognized as an anti-aging intervention, has also received abundant attention regarding SIRT3. At present, the impact of exercise on SIRT3 expression remains a topic for debate. The next two segments critically evaluate the evidence that exercise increases SIRT3 abundance in human and animal tissues.

## 3. The Effects of Exercise on SIRT3 Expression

### 3.1. Human Studies

Because of the importance of SIRT3 and its relation to exercise, numerous studies have investigated the effects of exercise on SIRT3 expression in human skeletal muscle and serum. Indeed, several reports conclude that exercise increases both muscular and serum SIRT3 levels in people of all ages [7,8,22]. However, controversy exists regarding the impact of different exercise modalities and duration on SIRT3 expression. Table 1 provides examples of the impact of numerous exercise modalities, intensities, and durations on SIRT3 in human muscle and serum.

Analysis of Table 1 reveals that acute exercise does not affect muscular SIRT3 expression in young or old people. For example, Edgett et al. reported that an acute bout of sprint-interval training in young adults does not impact SIRT3 protein expression in skeletal muscle [23]. Similar results have been reported by others [25]. Finally, Radak et al. investigated the acute effects of training in elderly people [24]. The results reveal that an acute bout of treadmill exercise failed to increase SIRT3 levels in aged individuals. Collectively, these studies indicate that acute exercise does not significantly impact muscular SIRT3 expression.

Further analysis reveals that short-duration exercise training (i.e., 3–6 weeks) does not increase SIRT3 abundance in humans. For example, Brandauer et al. reported that 3 weeks of one-legged knee extensor endurance training failed to elevate SIRT3 expression in skeletal muscle of young men [27]. Similarly, Edgett et al. concluded that 6 weeks of sprint interval training did not improve muscular SIRT3 levels in young adults [23]. 

In contrast to these negative findings, numerous studies have shown that regardless of age, chronic endurance exercise training (i.e., ≥8 weeks) is effective in improving SIRT3 expression in human skeletal muscle and serum. For instance, Johnson et al. [30] found that 8-week endurance training increased muscular SIRT3 levels in people aged 18–30 years and over 65 years. Similar findings were reported in two independent studies [29,31]. In these experiments, 12 weeks of aerobic exercise is proven sufficient in elevating muscular SIRT3 expression in adolescents. In line with these results, two studies have shown that 12 weeks of exercise is effective in enhancing SIRT3 levels in the serum of aged people (60 ± 6 and 66.23 ± 0.57) [32,33]. Moreover, Lanza et al. investigated the effects of four years of exercise training on aging-induced physiological dysfunction, including SIRT3 [7]. Importantly, this long-duration training program improved SIRT3 expression in both young (18–39 years) and old people (59–76 years). Lastly, Radak et al. investigated the SIRT3 expression in people who engaged in life-long exercise and reported that life-long physical activity can ameliorate aging-induced SIRT3 depression [8]. Together, these data show that 8 weeks or more of endurance training can effectively increase the expression of SIRT3 in skeletal muscle and serum in people of all ages. 

Compared to endurance training, only a limited number of studies have investigated the impact of resistance training on SIRT3 expression. A well-designed study in older people (66 ± 0.6 years) revealed that 12 weeks of resistance training (3 days/week) elevated SIRT3 expression [32]. In contrast, 12 weeks of resistance training (3 days/week) does not elevate SIRT3 levels in adolescents [29]. Similarly, 10 weeks of resistance training also failed to elevate the SIRT3 level in skeletal muscle of middle-aged people [28]. Collectively, these studies suggest the possibility that exercise-induced SIRT3 changes are protocol-dependent, and resistance training can ameliorate aging-induced SIRT3 suppression but does not improve SIRT3 levels in adolescents and middle-aged people. 

In summary, human studies indicate that exercise-induced SIRT3 changes are both time and exercise modality-dependent. Specifically, acute and short-duration endurance training (≤6 weeks) does not increase SIRT3 expression in human skeletal muscles. In contrast, long-duration endurance training (≥8 weeks) increases SIRT3 expression within skeletal muscles and serum in people of all ages. Although limited studies exist, the available evidence suggests the hypothesis that resistance training increases SIRT3 expression in muscles of older people but has limited effects on SIRT3 levels in young and middle-aged adults. Additional studies are required to fully understand the impact of different exercise modalities on SIRT3 expression in human tissues.

### 3.2. Rodent Studies

Because of ethical considerations, the investigation of exercise-induced SIRT3 expression in humans has been limited to serum and skeletal muscle. Preclinical studies do not suffer this limitation, and exercise's impact on SIRT3 expression has been investigated in numerous tissues. This is important because SIRT3 is also widely expressed in several tissues with high metabolic demand, including the brain, heart, kidney, cardiac muscle, and brown adipose tissue. Further, SIRT3 is also expressed in the reproductive system in males and females [34]. Table 2 summarizes animal studies investigating the impact of exercise training on SIRT3 expression in various rodent tissues. Please note that these studies include both acute and chronic exercise studies performed on healthy and diseased animals. Moreover, studies have incorporated both wild-type and knock-out animals. Collectively, these studies provide a clearer picture of the effects of exercise on SIRT3 expression in mammalian tissues.

Identical to human studies, acute bouts of exercise have limited effects on SIRT3 expression in rodents [22,35,36]. Moreover, animal studies have further verified the effects of acute exercise on intracellular organelles’ SIRT3 expression. Originally, Scher et al. proposed in 2007 that SIRT3 is transported from the nucleus to the mitochondria upon cellular stress [20]. However, the translocation of SIRT3 does not appear to respond to exercise in rodents because the mitochondrial SIRT3 levels are unchanged after exercise [36]. Importantly, rodent experiments investigated the effects of post-exercise recovery on SIRT3 expression. One time-point study by Gudiksen et al. reported that SIRT3 expression is not increased within the first 120 min following acute exercise in either wildtype and interleukin 6 (IL-6) knockout mice [35]. Notably, this study discovered that although metabolic signaling, including AMP-activated protein kinase (AMPK) and acetyl CoA carboxylase (ACC), increased upon exercise stimulation, muscular SIRT3 stayed at basic line levels 10, 60, and 120 min after exercise. 

While acute exercise does not alter SIRT3 abundance or expression, muscular SIRT3 levels are positively associated with exercise performance. For example, Vassilopoulos et al. reported that endurance exercise performance is decreased in SIRT3 knockout mice compared to wildtype [63]. Furthermore, studies using selectively bred high- and low-running-capacity rats have concluded that high-running-capacity rats have higher SIRT3 protein content in skeletal muscle compared to low running capacity; further, longer-lived high-running-capacity rats have higher SIRT3 in skeletal muscle [10]. Together, these results indicate that baseline SIRT3 level is linked to aerobic capacity and possibly interconnected with longevity.

Importantly, two independent studies conclude that chronic electrical stimulation of skeletal muscles in rodents increases SIRT3 abundance in skeletal muscle fibers [36,39]; these results reveal that skeletal muscle contractions stimulate the production of SIRT3. Moreover, prolonged muscle inactivity decreases SIRT3 expression in the inactive skeletal muscle [22]. Hokari et al. found that 4 weeks of hindlimb immobilization significantly suppressed SIRT3 expression compared with the free hindlimb. Together, these discoveries strongly support the idea that exercise upregulates SIRT3 levels by muscular contraction, whereas muscle inactivity results in decreases in SIRT3 expression. Interestingly, even though muscular contraction increases the expression of SIRT3, moderate exercise elicits more SIRT3 expression compared with high-intensity training. Studies investigating the impact of exercise intensity on SIRT3 expression reveal that compared with high-intensity interval training (HIIT), which strongly stimulates skeletal muscle contraction, endurance treadmill running, and voluntary running have better effects on SIRT3 expression in the liver of rodents [42]. This study indicates the possibility that the effect of exercise on SIRT3 expression is intensity independent. Further research is needed to clarify this controversial phenomenon.

Rodent studies also reveal that the effect of exercise on SIRT3 expression is tissue dependent. For example, only long-term regular exercise (≥12 weeks) increases the expression of SIRT3 in brain tissues. Numerous studies using short-duration training protocols (i.e., 1–12 weeks) have produced negative outcomes [37,41,48,55]. Short-duration training failed to increase SIRT3 expression in the hippocampus, cortex, striatum, and hippocampus mitochondrial in young and aged mice. Nevertheless, long-term regular exercise (≥12 weeks) has been proven to elevate SIRT3 content in brains. Marques et al. found that 12 weeks of treadmill running and voluntary running significantly increased SIRT3 content in the cortex of young rats [57], and 4 months of endurance training also elevated SIRT3 levels in aged CD-1 mice [60]. Based on these data, we assume that only long-term exercise (≥12 weeks) augments SIRT3 expression in the brain. Moreover, rodent experiments show that as little as 1 week of exercise can upregulate muscular SIRT3 expression, including cardiac and skeletal muscle in young and aged rodents. Accordingly, starting from 1-week interventions and ranging to months, numerous experiments have confirmed that different training protocols, including endurance training, voluntary running, HIIT, and resistance training, can increase muscular SIRT3 expression in rodents of all ages [22,27,40,44,50,51,53,54,59,61,64]. Of note, animal studies have proposed that exercise combining extra protein supplementation promotes exercise-induced SIRT3 elevation [61], and animals that underwent a maternal low-protein diet exhibit downregulated SIRT3 levels [64]. These experiments suggest that inadequate protein intake may explain some of the divergences in previous research findings.

In addition to muscle and brain, exercise also positively regulates liver SIRT3 content. One key study proved that 4 weeks of endurance treadmill running, voluntary running, and interval treadmill running can elevate SIRT3 expression in the liver of rats [42]. Based on this, Ascensão et al. discovered that 5 weeks of endurance training also increased SIRT3 in liver mitochondria [46]. Furthermore, longer periods of exercise of all forms produced the same outcomes in liver and liver mitochondrial [49,56,58]. 

Interestingly, long-term exercise training (≥1 year) studies have yielded some paradoxical results. For example, following 1 year of voluntary running, muscular SIRT3 content was not altered in rats with low or high running capacity [10]. Moreover, Nogueira et al. discovered that although one year of exercise prominently improved cardiac function of rodents, SIRT3 levels were not increased above sedentary controls [62]. At present, the mechanism(s) explaining why the exercise-induced increases in SIRT3 levels in skeletal muscles observed following 8–20 weeks of training are no longer present following 52 weeks of exercise remain poorly understood.

In conclusion, although some contradictory results exist, the bulk of the scientific literature indicates that 8–20 weeks of endurance exercise increases SIRT3 levels in the brain, liver, and skeletal muscle. The next segments of the review discuss the effects of elevated SIRT3 on aging. Briefly, aging-related hyperacetylation leads to mitochondrial dysfunction and oxidative damage. The increase in SIRT3 expression and its deacetylase activity may play a pivotal role in maintaining the balance between protein acetylation and deacetylation. 

## 4. The Effects of SIRT3 on Aging

Despite decades of research, the cellular mechanism(s) responsible for age-related cellular functional deterioration remains unclear. Although the pathological features of aging vary from tissue to tissue, a hallmark of cellular aging is mitochondrial dysfunction and oxidative damage to cellular components [65]. While the causes of age-related mitochondrial dysfunction and oxidative damage are likely multifactorial, a potential trigger is the increased protein acetylation in mitochondria during aging [66,67,68]. To begin with, studies conclude that age-related hyperacetylation induce mitochondrial dysfunction, represented by the defects in tricarboxylic acid (TCA) cycle, electron transport chain (ETC), and key transcription factors including the peroxisome proliferator-activated receptor gamma, coactivator 1 alpha (PGC-1α), nuclear factor kappa B (NFκB), and forkhead box O family TF (FOXO) [69,70]. As a result, the hyperacetylation of these enzymes and transcription factors leads to the decline of mitochondrial homeostasis and deteriorative diseases. Furthermore, aging is also accompanied by oxidative damage in cells leading to disturbed redox signaling [66]. Hyperacetylation is predicted to play an important role in this oxidative stress; for instance, acetylation of superoxide dismutase (SOD) 2 suppresses its catalytic activity and elevates superoxide anion levels in the mitochondrion thus leading to oxidative damage to lipids, protein, and mitochondrial DNA [71]. Therefore, maintaining cellular protein/deacetylation balance is critical for maintaining healthy cells [72]. In this regard, studies reveal that SIRT3 is the major mitochondrial deacetylase and plays a key role in preventing age-related mitochondrial dysfunction and oxidative stress by deacetylation [73].

SIRT3 was among the first genes identified to extend lifespan [74]. As early as 2003, it became recognized that SIRT3 was associated with longevity in humans [75]. These early studies triggered numerous investigations to determine the biological connection between SIRT3 expression and longevity. For example, studies reveal that mice lacking SIRT3 have significantly shortened lifespans [76] and spontaneously develop several aging-related diseases, including cancer, metabolic syndrome, cardiovascular disease, and neurodegenerative diseases [2]. Together, these studies reveal that SIRT3 plays a key role in the development of disease and aging. Clinical studies conclude that sedentary older people have nearly 40% reduced SIRT3 content compared with younger people, and high SIRT3 expression levels are associated with human longevity [75,77,78]. A major cause of the decline in SIRT3 activity is a decrease in NAD levels with age, and this decline is accelerated by sedentary and counteracted by caloric restriction and physical activity [71,79]. Additionally, growing evidence indicates that restoration of SIRT3 and NAD are potential therapeutic targets for aging [12,78]. Recently studies have established that SIRT3-induced counter-aging effects are primarily achieved through its deacetylase activity [13]. As discussed earlier, SIRT3 is the major mitochondrial deacetylase since SIRT3 knockout mice are plagued with a dramatic elevation in mitochondrial protein acetylation in a variety of tissues [20,80]. Indeed, SIRT3 can directly interact with at least 84 mitochondria proteins and many of these proteins are involved in important mitochondrial functions [81,82,83]. 

In summary, studies reveal that hyperacetylation plays an important role in aging-induced physiological dysfunction; this hyperacetylation results in impairments in both mitochondrial bioenergetics and redox balance. Notably, SIRT3, the main mitochondrial deacetylase protein, is well-recognized to be associated with longevity in animals and humans. The topic of the deacetylation impact of SIRT3 on both metabolism and redox balance will be addressed in more detail in the next section.

### 4.1. The Link between Deacetylase Activity of SIRT3 and Cellular Metabolism 

Metabolic changes are hallmarks of aging, as genetic and pharmacologic alterations of relevant pathways can extend life span [84]. One of the primary targets of SIRT3 deacetylase is metabolism-associated proteins. Studies using SIRT3 knockout mice suggest that SIRT3 has a prominent role in maintaining metabolism homeostasis [47,85,86,87]. In fact, SIRT3 is heavily involved in various physiological metabolic processes through deacetylation, and thus involved in the process of several diseases of aging, including cancer, heart diseases, metabolic diseases, etc. [1,2]. Table 3 demonstrates specific metabolic pathways regulated by SIRT3.

Importantly, SIRT3 plays a critical role in ETC [1]. The major function of SIRT3 in mitochondria is maintaining basal ATP levels through regulating the ETC by activating several ETC complexes through deacetylation, including complex (I–V) [1,73,89]. This is supported by the evidence that SIRT3 knockout mice have lower ATP levels in the heart, kidney, and liver [88]. Interestingly, SIRT3 function is regulated by complex I through the NAD+-SIRT3 pathway. As NADH is dehydrogenized to form NAD+ by complex I in the respiration chain, the resultant ratio of NAD+/NADH directly affects the activity of SIRT3 [102]. In this regard, the interaction between SIRT3 and complex I may be one of the crucial mechanisms. 

SIRT3 also functions in metabolic control by promoting fatty acid oxidation. SIRT3 deficient mice accumulate long-chain fatty acids and decrease fatty acid oxidation during calorie restriction [85]. Furthermore, 71% of the proteins involved in fatty acid oxidation increase acetylation in SIRT3 deficient mice’s liver mitochondria [103]. Mass spectrometry analysis revealed that SIRT3 targets numerous enzymes involved in fatty acid oxidation, such as long-chain acyl-CoA dehydrogenase (LCAD), medium chain-specific acyl-CoA dehydrogenase (MCAD), and very long-chain acyl-CoA dehydrogenase (VLCAD) [13,92,93].

In addition, SIRT3 is involved in protein metabolism. SIRT3-mediated deacetylation activates glutamate dehydrogenase 1 (GLUD1), the enzyme that produces α-ketoglutarate from glutamate and is involved in the TCA cycle and ammonia metabolism [93,96]. Additionally, through deacetylation, SIRT3 is involved in glutaminase (GLS) 2 activity, a primary enzyme responsible for glutamine catabolism [97,104]. Furthermore, as part of amino acid catabolism, SIRT3 was reported to deacetylases ornithine transcarbamylase (OTC) to regulate the second step of the urea cycle [99]. 

Moreover, controversial data indicate that SIRT3 participates in glucose metabolism and Warburg effects. As mentioned above, SIRT3 activates GLUD1 to promote glucose and ATP synthesis by enabling amino acids to be used as fuels. On the other hand, SIRT3 knockout contributes to the upregulated glycolysis but is companied with the downregulated mitochondrial metabolism [105]. One study hypothesis that reduced SIRT3 levels promote glycolysis via two mechanisms [93]. First, in the absence of SIRT3, the peptidylprolyl isomerase D (PPID) will be highly acetylated, activating hexokinase II (HK2), a key glycolytic enzyme. Consequently, HK2 phosphorylates glucose to produce glucose-6-phosphate (G6P), the first step in most glucose metabolism pathways [100,106]. Second, loss of SIRT3 increases reactive oxygen species (ROS) production, which stabilizes hypoxia-inducible factor (HIF) 1α, a transcription factor that regulates glycolytic gene expression [101].

SIRT3 also regulates ketone body production [94]. Ketone body production is regulated by 3-hydroxy-3-methylglutaryl CoA synthase-2 (HMGCS2) via multiple signals. SIRT3 can deacetylate HMGCS2 in response to metabolic stimulation [94]. Besides, SIRT3 can deacetylate the pyruvate dehydrogenase complex (PDC) to promote acetyl-CoA's conversion under normal conditions. Meanwhile, SIRT3 also facilitates the conversion of acetyl-CoA into ketone bodies to provide energy during fasting [94]. These studies suggest that SIRT3 plays a crucial role in the relation between glycolysis, Krebs cycle, and ketone body metabolism [107].

In summary, SIRT3 deacetylase various enzymes involved in multiple metabolic pathways, including glucose and ketone body metabolism, ATP production, fatty acid oxidation, and amino acid cycling, thus playing a pivotal role in balancing metabolic homeostasis. However, since there is a wide range of cascades as the downstream signing of SIRT3, no single protein target can fully explain the biological and physiological effects of SIRT3. Importantly, many of these targets are also associated with exercise, suggesting a potential exercise-sensing role of SIRT3 and metabolism [14,108]. 

### 4.2. Deacetylase Activity of SIRT3 Alters Redox Balance

SIRT3 also participates in the regulation of oxidative balance by deacetylation of substrates involved in both ROS production and detoxification. Several studies have demonstrated that the beneficial role of SIRT3 in aging and diseases are mainly due to its scavenging effect on ROS [89].

SIRT3 is involved in numerous antioxidant pathways. As mentioned above, SIRT3 can promote the effectiveness of electron transport via ETC complex components. Since ETC is one of the main producers of ROS, SIRT3 can reduce ROS production indirectly by regulating ETC efficiency [65]. By deacetylation, SIRT3 also directly regulates the activity of several superoxide scavengers to reduce superoxide production and protect against oxidative stress [65]. To begin with, SIRT3 regulates the activity of superoxide dismutase 2 (SOD2) through deacetylation. Knockout studies have shown that SIRT3 knockout rodents display an increase in mitochondrial superoxide production, which is associated with decreased SOD2 activity in the skeletal muscle, heart, and liver [109,110]. On the contrary, the upregulation of SIRT3 deacetylates SOD2 and enhances its activity, hence reducing mitochondrial oxidative stress [65]. Together, these results well-established the SIRT3-SOD2 relation in the redox balance. Furthermore, SIRT3 deacetylates and activates the TCA cycle enzyme isocitrate dehydrogenase (IDH) 2 and helps replenish the mitochondrial pool of NADPH, which is a key factor that affects glutathione reductase, a part of the antioxidant defense system against cellular oxidative stress [111]. 

In addition to the direct activation of antioxidant proteins, SIRT3 regulates the expression of antioxidant enzymes through the transcription factor FOXO3a and the transcriptional coactivator PGC-1α. Firstly, SOD2 and other antioxidant enzymes, like catalase, can be activated by SIRT3 via FoxO3 [112]. Studies discovered that SIRT3-FOXO3 induced signaling could significantly improve mitochondrial quantity and quality, which helps to preserve the mitochondrial capacity, thereby protecting cells from oxidative damage [112,113]. Second, SIRT3-dependent upregulation of PGC-1α is also involved in antioxidants [89]. Studies reported that PGC-1α regulates the expression of several mitochondrial antioxidant genes, including SOD2 and catalase, and thus prevents oxidative injury [89,114], and Sirt3 is essential for the PGC-1α-dependent induction of ROS-detoxifying enzymes [115].

Interestingly, SIRT3 not only limits the amount of mitochondrial ROS but also promotes the repair of oxidative DNA damage, especially in mitochondrial DNA, via the stabilization of 8-oxoguanine-DNA glycosylase 1 (OGG1), which repairs oxidative damage from DNA [116]. Under normal conditions, SIRT3 regulates redox balance and metabolism to maintain mitochondria homeostasis, influencing cell fitness and survival. However, when the mitochondria are too damaged to be repaired, SIRT3 can deacetylate and activate FOXO3a to induce mitophagy [1,117], thus contributing to mitochondrial renewal.

In summary, studies conclude that SIRT3 deacetylases various enzymes, directly and indirectly, to participate in redox balance. Thus, together with its relation with metabolism, SIRT3 is heavily involved in cellular metabolism, mitochondrial homeostasis, and redox balance. As aging-induced hyperacetylation also participated in these three processes, targeting SIRT3 is a promising therapeutic strategy in the prevention of pathological consequences of aging.

### 4.3. The Paradox between Aging and SIRT3 Levels

Accumulating evidence has documented that SIRT3 ameliorates many types of human diseases, including cancer, heart disease, and metabolic disorder, indicating SIRT3 is a potential therapeutic target [1]. Zhang et al. have systematically reviewed the possible mechanisms of SIRT3 in numerous diseases and aging [1]. However, even though numerous works have confirmed that SIRT3 is involved in aging, some rodent studies indicate contradictive data (Table 4). This section will discuss these paradoxes and try to explain the mechanism.

With regard to humans, studies have established that SIRT3 expression is decreased with age. For example, Radak et al. report that SIRT3 levels significantly decline in skeletal muscle of senescent adults [24]. In addition, Matthew et al. report that aging significantly suppressed SIRT3 levels and antioxidant proteins in the skeletal muscle of aged people [30]. Furthermore, Lanza et al. reported that in sedentary people, mitochondrial function and SIRT3 expression are lower with aging [7]. However, data from rodent studies do not agree with these findings. Although several rodent studies reported that SIRT3 is negatively correlated with aging in several tissues, and aging-induced SIRT3 suppression is accompanied by reductions in mitochondrial health (elevated ROS production, reduced markers of mitochondrial biogenesis, and disturbance of energy production) [43,53,60], some experiments report that SIRT3 levels remain unchanged or increased in aged rodents. Interestingly, Karvinen et al. using high- and low-running-capacity rats, found that high SIRT3 level is related to longer lifespan in rats, but aging itself is not connected with SIRT3 level [10]. Surprisingly, Dongwook reported that aged muscles demonstrated clear signs of mitochondrial dysfunction, oxidative stress, and inflammation with increased acetylation; but SIRT3 is unexpectedly increased [70]. Radak et al. Additionally confirmed the increase of SIRT3 and hyperacetylation during aging in the brain of rats [48]. These studies raise the question of why SIRT3, as the main deacetylase factor, increases in aged rodents with increased acetylation conditions. Notably, it is important to highlight that this paradox is only reported in rodents, while the majority of human studies indicate that SIRT3 is negatively associated with aging and aging-induced physiological declines.

Based on these results, one study assumes that NAD+ is indispensable for SIRT3 [70,72]. The insufficient supply of NAD+ may be a limiting factor in intracellular deacetylation capacity despite increased SIRT expression. Vivo studies have shown that SIRT3 activity can be severely hampered due to diminished NAD+, therefore resulting in acetylation of key enzymes and transcription factors, causing mitochondrial dysfunction and redox disturbance [70,72]. On the other hand, SIRT3 is also indispensable for NAD-induced beneficial effects. Pillai et al. argued that NAD+ treatment-induced cardiac protective effects disappeared in SIRT3 knockoff mice [119], suggesting the complementary effects of SIRT3 and NAD+. Although the specific mechanism is still undetermined, current results have shown that SIRT3 and NAD+ are essential to each other.

Together, current studies propose the importance of NAD in the signaling of SIRT3. Not only limited to the activator of expression but also interconnected with the physiological function of SIRT3, including aging. Hence, exercise, as an effective intervention modulating NAD level and SIRT3 expression together, deserve more attention. 

## 5. The Effects of SIRT3 in Exercise-Induced Adaptation

As mentioned above, SIRT3 and its cascade regulate aging via maintaining metabolism and redox balance homeostasis. Additionally, studies have proven that exercise is positively associated with SIRT3 expression, with numerous health benefits [7]. Correct forms of training elevate SIRT3 levels in people of all ages and ameliorate aging and disease-induced SIRT3 suppression. Next, we summarize information in support of connections of SIRT3 with exercise-induced metabolic and oxidative changes.

### 5.1. The Role of SIRT3 in Exercise-Induced Metabolic Changes

Exercise, one of the most well-studied metabolic interventions, has long been considered beneficial to healthspan and lifespan extension via metabolic stimulation in multiple models, ranging from animals to humans [120]. Since SIRT3 is also involved in aging and metabolic control, and exercise is positively associated with SIRT3, here we will discuss whether SIRT3 is involved in exercise-induced metabolic changes.

First, SIRT3 plays a pivotal role in muscular metabolic ability. It is well known that SIRT3 is highly expressed in tissues with high metabolic demand, including skeletal muscle, and one of the primary functions of SIRT3 is directly regulating several metabolism pathways [13]. As early as 2009, one study found that SIRT3 is highly expressed in slow oxidative type I muscle compared to fast type II muscles [40], in accordance with higher mitochondrial content and the oxidative feature of type I muscle. Additionally, in skeletal muscle, SIRT3 appears to localize solely to mitochondria, which is the factory of ATP production during exercise [36], and its expression scales with markers of skeletal muscle oxidative capacity, including cAMP-response element-binding protein(CREB), citrate synthase (CS), PGC-1α and AMPK [36,40]. Based on these results, we conclude that SIRT3 expression is positively related to metabolic ability and energy production in skeletal muscle.

Importantly, numerous studies have proposed that SIRT3 is involved in exercise-induced metabolic changes [14]. To begin with, ATP synthase contains multiple SIRT3-dependent reversible acetyl-lysines, which are altered in several conditions of metabolic stress, including exercise [63]. One critical study has suggested the possibility that SIRT3 is the critical regulatory enzyme that generates ATP in response to ATP demand during exercise [11]. This study concluded that exercise-induced excessive demand for ATP will cause an increase in the NAD+ content and, therefore, NAD+/NADH ratio to provide oxidoreductive power for the generation of ATP by mitochondria. Consequently, the disturbed NAD+/NADH ratio activates SIRT1 and SIRT3. As mentioned above, NAD+ is essential to the activity of SIRT3; therefore, the increased NAD+ during exercise will promote the deacetylase effects of SIRT3 [70,72,119]. Then, the increased SIRT3 activity will prompt the production of ATP via its deacetylates effects to activate the TCA cycle, ETC, and β-oxidation [1,73]. This is supported by the evidence that mice lacking SIRT3 show decreased muscle endurance upon exercise stimulation [63], and a higher SIRT3 level is associated with more efficient ATP production in skeletal muscle and consequently increases exercise performance in high running capacity subjects [121]. Additionally, one in vitro study verified that the knockdown of SIRT3 in C2C12 muscle cells decreases basal and maximal oxygen consumption rates and prevents PGC1α-induced activation of mitochondrial genes [115,122]. These studies strongly support the idea that SIRT3 is a key regulator in exercise-induced metabolism changes. However, whether SIRT3 directly controls the ATP production in skeletal muscle during exercise is not verified. Future studies are needed to explore the specific mechanism further. 

Interestingly, even though SIRT3 is involved in energy production, studies demonstrate that SIRT3 itself may act to suppress mitochondrial protein synthesis and thus mitochondrial biogenesis. Yang’s study revealed that SIRT3 negatively regulates the activity of mitochondrial ribosomes and the knockdown of SIRT3 in C2C12 cells enhanced mitochondrial protein synthesis via SIRT3 dependent deacetylate mitochondrial ribosomal protein L10 (MRPL10) [98]. Therefore, SIRT3 seems to suppress mitochondrial protein synthesis in skeletal muscle. Based on this, one study assumes that during exercise, it is necessary to generate ATP to maintain force production, so pathways that utilize energy, such as protein synthesis, would be temporarily suppressed; therefore, the actions of SIRT3 are akin to the effects of AMPK on enhancing energy production and muscle work, while inhibiting pathways that use energy [11]. Because of these unclear effects, it will be of interest to investigate in detail in future studies. For example, whether exercise-induced mitochondrial biogenesis and energy production is impaired in SIRT3-null mice.

While SIRT3 has been shown to reduce mitochondrial protein synthesis, which maximizes ATP production, numerous studies have proposed that SIRT3 contributes to mitochondrial biogenesis through PGC1α-dependent and independent mechanisms [11,115]. PGC-1α is well known for its relations with mitochondria and is regarded as the classic mitochondrial biogenesis biomarker. One in vitro study using C2C12 myotubes discovered that SIRT3 is involved in several physiological functions of PGC-1α [115]. Firstly, they proposed that PGC-1α strongly stimulates SIRT3 gene expression in muscle and the knockdown of PGC-1α led to decreased SIRT3 gene expression. In addition, the study indicated that SIRT3 knockdown decreases the stimulatory effect of PGC-1α on mitochondrial biogenesis and ROS-detoxifying enzymes in C2C12 myotubes [115]. Meanwhile, animal studies produced the same outcome. Gurd et al. discovered that rodent muscular SIRT3 level is proportional to PGC-1α [36], and SIRT3 KO mice were found to suffer from hampering of mitochondrial function in the heart with the sign of aging [123]. Based on these contradictive data, one study proposed one assumption that during exercise, SIRT3 reduces mitochondrial protein synthesis, thus maximizing the availability of ATP production; after exercise, SIRT3 may be involved in mitochondrial adaptation [11]. However, the specific mechanism is still unclear. 

Taken together, despite some uncertain data, studies have proven that SIRT3 plays an important role in regulating mitochondrial biogenesis, energy production, and homeostasis in skeletal muscle. Based on current results, researchers have made assumptions that the role of SIRT3 during and after exercise is different. However, direct evidence is still needed to verify the metabolic relation between SIRT3 and exercise.

### 5.2. The Role of SIRT3 in Exercise-Induced Antioxidant Adaptation

Increased oxidative damage is a major hallmark of aging. In the last century, countless types of research have contributed to exploring the area of oxidative stress in aging and disease individuals. Exercise, as one of the most efficient interventions to suppress the process of aging and upregulate antioxidant capacity, has received numerous attention for its effects on maintaining redox balance [124]. Regular exercise has long been known to upregulate antioxidant enzymes in response to metabolic activity-induced oxidative stress [125]. As mentioned earlier, studies conclude that SIRT3 plays a critical role in redox balance, and appropriate modes and durations of exercise are positively related to SIRT3 expression in subjects of different ages. 

Previous human studies have demonstrated that exercise can counteract aging-induced oxidative stress by activating antioxidant proteins, for example, SOD2, and this effect is positively related to SIRT3 expression [8,30]. Radak et al. discovered that life-long exercise could ameliorate aging-induced SOD2 and SIRT3 suppression in human skeletal muscle [8]. Furthermore, Johnson et al. demonstrated that aging significantly suppressed the expression of SOD2 and SIRT3 in elderly people, and 8 weeks of endurance training can increase the activity of SOD2 and SIRT3 expression in skeletal muscle [30]. In addition, rodent studies also proved this phenomenon and further explored this effect in different tissues, including skeletal muscle [115], brain [57], liver [58], and heart [59]. Hence, the effects of exercise-induced elevation of SIRT3 and antioxidant proteins are proved in various organs. Additionally, some rodent experiments assume that the exercise-induced SIRT3 elevation can ameliorate diseases by regulating redox stress. This relation is found in the obesity model [47], myocardial infarction model [51], APP/PS1 Transgenic induced cognition disorder model [55], sporadic inclusion body myositis [52], and spontaneously hypertensive model [59]. Nevertheless, the specific pathway is unclear because these studies did not use inhibitors.

Importantly, an animal study confirmed that AMPK is required to upregulate SIRT3 and SOD2 protein levels in response to exercise training. This study found that 6 weeks of exercise increased protein expression of SIRT3 and SOD2 in the skeletal muscle of mice; however, this effect disappeared in AMPK-α2 knock-off mice [27]. Based on these results, this study also tested the effects of chronic AMPK activator treatment on mice’s skeletal muscle. They reported that 4 weeks of AMPK activator itself significantly elevated SIRT3 and SOD2 protein and mRNA expression, suggesting the possibility that AMPK is involved in exercise-induced SIRT3 elevation and antioxidant effects. To further test the signaling cascade, this study used AMPK activator in mice lacking PGC-1a expression. They indicate that AMPK activator-induced adaptation disappeared in mice lacking PGC-1a expression. Together, this key study suggests that SIRT3 and SOD2 protein expression is regulated in a signaling axis involving both AMPK and PGC-1α [27]. Another cell study also proposes the importance of PGC-1α in SIRT3-induced redox balance [115]. They concluded that the overexpression of SIRT3 or PGC-1α in C2C12 myotubes decreased basal ROS levels. In contrast, knockdown of SIRT3 increased basal ROS level and blocked the inhibitory effect of PGC-1α on cellular ROS production, suggesting SIRT3 mediates the PGC1α-induced antioxidant effects in muscle cells [115]. Together, rodent and in vitro studies have clearly shown that AMPK and PGC-1α are key regulators in exercise-induced SIRT3 elevation and, consequently, the increase of antioxidant proteins. However, one study indicates the opposite result. They reported that 5 days of AMPK activator failed to elevate SIRT3 level but increased PGC-1α content and mitochondrial network in skeletal muscle [36]. Therefore, this study and subsequent articles questioned whether AMPK and PGC-1α are necessary factors in exercise-induced SIRT3 changes. Based on our collected evidence, we assume that, similar to exercise experiments, acute or short-term AMPK intervention is not effective in changing SIRT3 levels [27,36].

Another key study using SIRT3-knockout mice proposed the possibility that SIRT3-SOD2 is involved in exercise-triggered cognitive function improvement [47]. Their findings confirmed that the antioxidant effect of the SIRT3-SOD2 pathway is critical for the survival of neurons. Firstly, they demonstrated that high-fat diet (HFD) induces the suppression of SIRT3 and acetylated inactivation of SOD2 in the hippocampus, thus increasing the levels of oxidative stress and damaging cognitive function. Notably, SIRT3 deficiency markedly exacerbated the oxidative stress and cognitive damage after HFD treatment. Nevertheless, aerobic interval training attenuated neuronal apoptosis and improved cognitive function in mice with HFD through the positive regulation of the SIRT3-SOD2 pathway and the reduction of oxidative stress levels. This critical finding placed the SIRT3-SOD2 pathway as a new factor in the neuroprotective field.

In the end, it is important to mention that although current studies have established the positive relationship between exercise and the SIRT3-SOD2 pathway, some experiments discovered that these two factors are not bonded in certain circumstances. For example, Santos [58] proposed that 12 weeks of endurance training increased SIRT3 levels in the liver but has no effect on SOD2 expression. Meanwhile, Radak [9,126] suggested that 20 weeks of interval training significantly ameliorated the oxidative stress in the liver of APP/PS1 transgenic mice; however, the SIRT3 level did not change. Furthermore, another one-year rodent training program discovered the elevated SOD2 expression in the right ventricle, but SIRT3 did not change after this super-long training [62]. These studies indicate that exercise-induced adaptation is more sophisticated, so SIRT3 itself only plays part of the role. As reviewed in this article, one potential mechanism may be the changes in the NAD+ level.

Based on current human and rodent evidence, we propose that exercise-induced SIRT3 changes are positively associated with SOD2 in skeletal muscle, brain, liver, and heart. Additionally, exercise-induced SIRT3 changes can ameliorate disease conditions via its antioxidant effects. Furthermore, strong evidence has suggested that AMPK and PGC-1α are involved in exercise-induced SIRT3 changes and follow-up antioxidant effects. In conclusion, despite some conflicting research, SIRT3 plays a positive role in exercise-induced antioxidant adaptation via AMPK and PGC-1α.

## 6. Summary

This review systematically evaluated the current knowledge of SIRT3 and its relationship with aging and exercise. Through deacetylation, SIRT3 provides various anti-aging benefits ranging from maintaining metabolic homeostasis to protecting redox balance. Based on current evidence, it is conceivable that controlling aging-induced SIRT3 decline helps preventing disorders of body functions. As reviewed in this work, regardless of age and gender, regular exercise positively affects SIRT3 expression in multiple tissues and disease conditions. Specifically, exercise can ameliorate aging-induced SIRT3 suppression. Thus, we propose regular exercise as an effective intervention to improve SIRT3 expression and therefore, alleviate aging-induced disorders. Moreover, this article evaluated the role of SIRT3 in exercise-induced adaptation. We concluded that the beneficial effect of exercise is partly mediated through SIRT3 via its deacetylation effects on metabolic and oxidative signaling. Special attention should also be placed on the interplay between SIRT3 and NAD+, as current results have shown that SIRT3 and NAD+ are essential to each other. Besides, more direct research is needed to elucidate the role of SIRT3 during and after exercise to confirm if these benefits apply to all populations and disease conditions. Answering these questions will enhance our understanding of the mechanism of exercise-induced adaptation and promote the development of therapeutic methods for aging.

## Figures and Tables

**Table 1:** The effects of exercises on SIRT3 expression in human studies.

Exercise Protocol	SIRT3 Protein Level	Age	Gender	Tissue	Reference
Acute Sprint Interval Training	Unchanged	22.0 ± 2.4	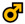	Skeletal Muscle	[23]
Acute Treadmill Running	Unchanged	63.4 ± 4.7	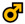	Skeletal Muscle	[24]
Acute Stationary Biking	Unchanged	21.4 ± 2.8	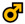	Skeletal Muscle	[25]
Acute Sprint Interval Swimming	Unchanged	23 ± 3	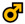	Skeletal Muscle	[26]
3 Weeks One-Legged Knee Extensor	Unchanged	Young Age	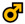	Skeletal Muscle	[27]
6 Weeks Sprint-Interval Training	Unchanged	22.0 ± 2.4	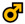	Skeletal Muscle	[23]
10 Weeks Resistance Training	Unchanged	Middle-Aged	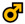 and 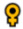	Skeletal Muscle	[28]
12 Weeks Resistance Training	Unchanged	16.7 ± 0.9	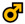	Skeletal Muscle	[29]
8 Weeks Endurance Training	Increased	18–30 and ≥65	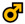 and 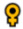	Skeletal Muscle	[30]
12 Weeks Endurance Training	Increased	16.7 ± 0.9	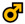	Skeletal Muscle	[29]
12 Weeks Endurance Training	Increased	15.5 ± 0.8	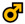	Skeletal Muscle	[31]
12 Weeks Resistance Training	Increased	66.23 ± 0.57	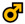	Serum	[32]
12 Weeks of Resistance and Endurance Training	Increased	60 ± 6	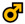 and 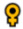	Serum	[33]
4 Years of Endurance Training	Increased	18–30 and 59–76	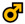 and 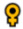	Skeletal Muscle	[7]
Life-Long Physical Activity	Increased	65 ± 5	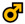 and 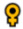	Skeletal Muscle	[8]

**Table 2:** The effects of exercises on SIRT3 expression in rodent studies.

Species	Gender	Training Period	Exercise Protocol	SIRT3 Content	Tissue	Reference
Wistar Rats	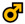	Acute	Endurance Treadmill Running	Protein Unchanged	Skeletal Muscle	[22]
C57BL/6 and IL-6 Knockout Mice	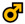	After Acute for 10, 60, 120 min	Endurance Treadmill Running	Protein Unchanged	Skeletal Muscle	[35]
Sprague Dawley (SD) Rats	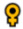	After Acute for 0, 180 min	Treadmill Running Until Exhaustion	Protein Unchanged	Muscular Mitochondrial	[36]
C57BL/6 Mice	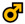	5 Days	Endurance Treadmill Running	Protein Unchanged	Brain	[37]
C57BL/6 Mice	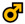	7 Days	Voluntary Running	mRNA Unchanged	Heart	[38]
SD Rats	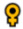	7 Days	Electrodes Stimulation	Protein Increased	Skeletal Muscle	[36]
SD Rats	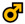	7 Days	Electrodes Stimulation	Protein Increased	Skeletal Muscle	[39]
Rats	Unknown	1 and 4 Weeks	Endurance Treadmill Running	Protein Increased	Skeletal Muscle	[40]
Young and Aged C57BL/6 Mice	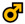	3 Weeks	Endurance Treadmill Running	Protein Unchanged	Brain	[41]
SD Rats	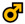	4 Weeks	Endurance Treadmill Running, Voluntary Running, Interval Treadmill Running	Protein Increased	Liver	[42]
Aged SD Rats	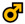	4 Weeks	Endurance Treadmill Running	Protein mRNA Increased	Nigral Region	[43]
SD Rats	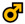	4 Weeks	Endurance Treadmill Running	Protein Increased	Muscular Mitochondrial	[44]
Wistar Rats	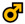	4 Weeks	Endurance Treadmill Running and Voluntary Running	Protein Increased	Skeletal Muscle	[22]
SIRT3 Overexpression Mice	Unknown	30 Days	Voluntary Running	Protein mRNA Increased	Brain	[45]
Wistar Rats	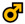	5 Weeks	Endurance Treadmill Running	Protein Increased	Liver Mitochondrial	[46]
C57BL/6 Mice	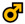	6 Weeks	Voluntary Running	Protein Increased	Skeletal Muscle	[40]
High Fat Diet-induced Obese Mice	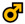	6 Weeks	Treadmill Intermittent Running	Protein Increased	Hippocampus	[47]
Young and Aged Wistar Rats	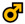	6 Weeks	Endurance Treadmill Running	Protein Unchanged	Hippocampus	[48]
Wild-Type Mice	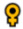	6.5 Weeks	Voluntary Running	Protein Increased	Skeletal Muscle	[27]
AMPK-KO Mice	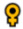	6.5 Weeks	Voluntary Running	Protein Unchanged	Skeletal Muscle	[27]
Healthy Rats	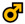	8 Weeks	Continuous and HIIT Swimming Training	Protein Increased	Liver	[49]
Wistar Rats	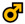	8 Weeks	Endurance Treadmill Running	Protein Increased	Heart Serum	[50]
Myocardial Infarction SD Rats	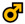	8 Weeks	HIIT Treadmill Running	Protein Increased	Heart	[51]
Sporadic Inclusion Body Myositis Wistar Rats	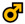	9 Weeks	Ladder Climbing	Protein Increased	Skeletal Muscle	[52]
Aged C57BL/6 Mice	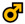	10 Weeks	Voluntary Running	Protein Increased	Arterial	[53]
SD Rats	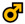	10 Weeks	HIIT and Endurance Treadmill Running	Protein Increased	Skeletal Muscle	[54]
SD Rats	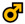	10 Weeks	HIIT and Endurance Treadmill Running	Protein Unchanged	Heart	[54]
APP/PS1 Transgenic Mice	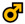	12 Weeks	Endurance Treadmill Running	Protein Increased	Hippocampus Mitochondria	[55]
Wild-Type Mice	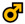	12 Weeks	Endurance Treadmill Running	Protein Unchanged	Hippocampus Mitochondria	[55]
SD Rats	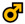	12 Weeks	Endurance Treadmill Running and Voluntary Running	Protein Increased	Liver Mitochondria	[56]
SD Rats	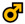	12 Weeks	Endurance Treadmill Running and Voluntary Running	Protein Increased	Brain	[57]
SD Rats	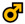	12 Weeks	Endurance Treadmill Running and Voluntary Running	Protein Increased	Liver	[58]
Spontaneously Hypertensive Rats	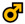	16 Weeks	Endurance Treadmill Running	Protein Increased	Heart	[59]
Aged CD-1 Mice	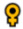	16 Weeks	Endurance Treadmill Running	Protein Increased	Brain	[60]
Low Running Capacity Rats	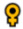	20 Weeks	Voluntary Running	Protein Increased	Skeletal Muscle	[61]
APP/PS1 Transgenic Mice	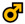	20 Weeks	Interval Treadmill Running	Protein Unchanged	Liver	[9]
Low and High Running Capacity Rats	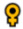	52 Weeks	Voluntary Running	Protein Unchanged	Skeletal Muscle	[10]
SD Rats	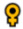	52 Weeks	Endurance Treadmill Running	Protein Unchanged	Heart	[62]

**Table 3:** Metabolic targets of SIRT3.

Function	Targets
Electron Transport Chain	Complex I [88,89] Complex II [90] Complex III [91] Complex IV [89] Complex V [73,89]
Fatty Acid Oxidation	LCAD [85] MCAD [13] VLCAD [92,93]
Ketone Body Synthesis	HMGCS2 [94]
Acetate Metabolism	ACSS2 [95]
Amino Acid Catabolism	GLUD1 [93,96] GLS2 [97] MRPL10 [98]
Urea Cycle	OTC [99]
Glycolysis	PPID [100] HIF1α [101] GLUD [93,96]

**Table 4:** The effects of aging on SIRT3 expression.

Species	Age	SIRT3 Content	Tissue	Reference
Human				
Human	63.4 ± 4.7	Decreased	Skeletal Muscle	[24]
Human	≥65	Decreased	Skeletal Muscle	[30]
Human	59–76	Decreased	Skeletal Muscle	[7]
Rodents				
Male C57BL/6 Mice	25 months	Decreased	Heart	[53]
Female CD-1 Mice	14 months	Decreased	Brain	[60]
Male SD Rats	18–20 months	Decreased	Nigral Region	[43]
Male Wistar Rats	22 months	Unchanged	Heart, Kidney, Plasma	[118]
Female High and Low Running Capacity Rats	21 months	Unchanged	Skeletal Muscle	[10]
Female C57BL/6 Mice	24 months	Increased	Skeletal Muscle	[70]
Male Wistar Rats	26 months	Increased	Hippocampus	[48]
